# Childhood and Adolescent Depression Symptoms and Young Adult Mental Health and Psychosocial Outcomes

**DOI:** 10.1001/jamanetworkopen.2024.25987

**Published:** 2024-08-08

**Authors:** Lamprini Psychogiou, Marie C. Navarro, Massimiliano Orri, Sylvana M. Côté, Marilyn N. Ahun

**Affiliations:** 1 Mood Disorders Centre, University of Exeter, Exeter, United Kingdom; 2 Department of Public Health, Bordeaux Population Health Research Centre, Institut National de la Santé et de la Recherche Médicale U1219, Bordeaux, France; 3 McGill Group for Suicide Studies, Douglas Mental Health University Institute, Department of Psychiatry, McGill University, Montréal, Quebec, Canada; 4 Department of Epidemiology, Biostatistics and Occupational Health, School of Population and Global Health, McGill University, Montréal, Quebec, Canada; 5 Department of Social and Preventive Medicine, Université de Montréal School of Public Health, Montréal, Quebec, Canada; 6 Axe Cerveau et Développement de l’Enfant, Centre Hospitalier Universitaire Sainte-Justine, Montréal, Quebec, Canada; 7 Department of Medicine, Faculty of Medicine and Health Sciences, McGill University, Montréal, Quebec, Canada; 8 Department of Global Health and Population, Harvard T.H. Chan School of Public Health, Boston, Massachusetts

## Abstract

**Importance:**

Depression is a leading cause of disability. The timing and persistence of depression may be differentially associated with long-term mental health and psychosocial outcomes.

**Objective:**

To examine if depression symptoms during early and middle childhood and adolescence and persistent depression symptoms are associated with impaired young adult outcomes independent of early risk factors.

**Design, Setting, and Participants:**

Data for this prospective, longitudinal cohort study were from the Québec Longitudinal Study of Child Development, a representative population-based Canadian birth cohort. The cohort consists of infants born from October 1, 1997, to July 31, 1998. This is an ongoing study; data are collected annually or every 2 years and include those ages 5 months to 21 years. The end date for the data in this study was June 30, 2019, and data analyses were performed from October 4, 2022, to January 3, 2024.

**Exposures:**

Depression symptoms were assessed using maternal reports in early childhood (ages 1.5 to 6 years) from 1999 to 2004, teacher reports in middle childhood (ages 7 to 12 years) from 2005 to 2010, and self-reports in adolescence (ages 13 to 17 years) from 2011 to 2015.

**Main Outcomes and Measures:**

The primary outcome was depression symptoms at age 20 years, and secondary outcomes were indicators of psychosocial functioning (binge drinking; perceived stress; not being in education, employment, or training; social support; and experiencing online harrasment) at age 21 years. All outcomes were self-reported. Adult outcomes were reported by participants at ages 20 and 21 years from 2017 to 2019. Risk factors assessed when children were aged 5 months old were considered as covariates to assess the independent associations of childhood and adolescent depression symptoms with adult outcomes.

**Results:**

The cohort consisted of 2120 infants. The analytic sample size varied from 1118 to 1254 participants across outcomes (56.85% to 57.96% female). Concerning the primary outcome, adjusting for early risk factors and multiple testing, depression symptoms during adolescence were associated with higher levels of depression symptoms (β, 1.08 [95% CI, 0.84-1.32]; *P* < .001 unadjusted and Bonferroni adjusted) in young adulthood. Concerning the secondary outcomes, depression symptoms in adolescence were only associated with perceived stress (β, 3.63 [95% CI, 2.66-4.60]; *P* < .001 unadjusted and Bonferroni adjusted), while both middle-childhood (β, −1.58 [95% CI, −2.65 to −0.51]; *P* = .003 unadjusted and *P* < .001 Bonferroni adjusted) and adolescent (β, −1.97 [95% CI, −2.53 to −1.41]; *P* < .001 unadjusted and Bonferroni adjusted) depression symptoms were associated with lower levels of social support. There were no associations for binge drinking; not being in education, employment, or training; or experiencing online harrasment.

**Conclusions and Relevance:**

In this cohort study of Canadian children and adolescents, childhood and adolescent depression symptoms were associated with impaired adult psychosocial functioning. Interventions should aim to screen and monitor children and adolescents for depression to inform policymaking regarding young adult mental health and psychosocial outcomes.

## Key Points

**Question:**

Are depression symptoms during childhood and adolescence associated with poor mental health and psychosocial outcomes in young adulthood?

**Findings:**

In this cohort study using a representative population-based Canadian birth cohort of 2120 infants, depression symptoms during adolescence (ages 13 to 17 years) were associated with higher levels of depression symptoms and perceived stress in early adulthood (at ages 20 and 21 years), while both middle-childhood (ages 7 to 12 years) and adolescent depression symptoms were associated with decreased social support for participants at age 21 years, independent of early risk factors. There were no associations of depression symptoms with binge drinking; not being in education, employment, or training; or experiencing online harrasment.

**Meaning:**

The findings of this study underscore the importance of screening children and adolescents for depression, which may reduce depression symptoms and compromised psychosocial functioning in young adulthood.

## Teaser

This cohort study assesses whether childhood and adolescent depression symptoms are associated with poor mental health and psychosocial outcomes in young adulthood.

## Introduction

Depression is a leading contributor to global disease burden.^1^ A nationally representative US study of 2016 data found that 3.2% of children and adolescents (ages 3 to 17 years) were depressed and that prevalence rates tended to increase with age.^2^ The timing of depression onset and symptom persistence may differentially impact an individual’s functioning. Longitudinal and meta-analytic evidence suggest that depression symptoms during adolescence are associated with mental health problems and impaired functioning in adulthood.^3,4,5,6,7^

Because available studies do not often examine depression symptoms during childhood, it is not yet clear whether symptoms occurring during early (ages 1.5 to 6 years) and middle (ages 7 to 12 years) childhood and adolescence (ages 13 to 17 years) are independently associated with adult mental health and psychosocial outcomes. Additionally, focusing on 1 developmental period precludes the examination of whether individuals with persistent symptoms are at higher risk for worse outcomes later in life. This omission has implications for prioritizing the allocation of support to individuals who are most at risk.^8^ Moreover, most studies focus on mental health as the primary outcome, thus overlooking the association of depression symptoms with pertinent psychosocial outcomes.^9^ Therefore, it is important to examine a broad range of outcomes to understand the associations of depression symptoms with overall functioning in adulthood to inform policymaking.^9^

Previous studies have investigated the associations of the timing of depression symptoms with adult outcomes.^8,10^ A study examining trajectories of depression symptoms from ages 10.5 to 25 years found that individuals with persistent early-onset depression symptoms during adolescence were associated with poorer mental health and work and educational outcomes in early adulthood.^8^ Another study found that depression during childhood and adolescence was associated with physical and mental health problems, risky behaviors, and problems in psychosocial functioning in adulthood.^10^ Importantly, individuals who had adolescent-onset vs childhood-onset depression and individuals with depressive symptomatology across childhood and adolescence had worse outcomes in adulthood.^10^

A limitation of the existing literature is that studies have often not considered a broad range of confounding factors.^10^ Several factors, including being female, having a limited-income background, being exposed to parental psychopathology, and experiencing problematic family relationships, are known risk factors for depression symptoms and impaired adult functioning.^11,12,13,14,15^ Therefore, it is important to consider these and other confounding factors to obtain an accurate estimate of the associations of childhood and adolescent depression symptoms with adult outcomes.^10^

The objective of this study was to examine the associations of depression symptoms in early and middle childhood with depression symptoms (primary outcome) and psychosocial outcomes (secondary outcome) in young adulthood. Our hypothesis for the current study was that childhood and adolescent depression symptoms would be associated with primary and secondary outcomes in early adulthood, but no a priori hypotheses were made about the associations of childhood and adolescent depression symptoms on any specific adult outcome.

## Methods

Data for this cohort study were drawn from the ongoing Québec Longitudinal Study of Child Development (QLSCD), a large, representative population-based birth cohort conducted by the Institut de la Statistique du Québec in Canada. The cohort in the QLSCD consisted of 2120 infants born from October 1, 1997, to July 31, 1998 (see the cohort profile for more information on the overall cohort^16^). The end date for the data in this study was June 30, 2019. Baseline characteristics were assessed when children were aged 5 months old by trained research assistants during interviews held at participants’ homes or using mailed questionnaires. Depression symptoms during early childhood (ages 1.5 to 6 years) were reported by children’s mothers, from 1999 to 2004 and during middle childhood (ages 7 to 12 years) by teachers, from 2005 to 2010, whereas adolescent depression symptoms were self-reported by participants at ages 13, 15, and 17 years from 2011 to 2015. Adult outcomes were reported by participants at ages 20 and 21 years from 2017 to 2019 using online questionnaires. Informed written consent was obtained by all participating families (and teachers) at each assessment point. Participants consented to data collection from age 18 years onward. Ethics were approved by the health research ethics committees of the Institut de la Statistique du Québec and the Sainte-Justine Hospital Research Centre. This study adhered to the Strengthening the Reporting of Observational Studies in Epidemiology (STROBE) reporting guideline for standard reporting in cohort studies.^17^

### Outcomes and Measures

The primary outcome of the present study was depression symptoms assessed at age 20 years. The secondary outcomes were indicators of psychosocial functioning (binge drinking; perceived levels of stress; not being in education, employment, or training [NEET] status; social support; and experiencing online harrasment at age 21 years.

We examined the independent and joint associations of depression symptoms in early and middle childhood and adolescence with young adult outcomes. Based on prior evidence, a broad range of covariates were adjusted for in the analyses. The early-childhood depression symptoms were reported when children were 1.5, 2.5, 3.5, 4.5, and 5, and 6 years of age, and middle-childhood symptoms when children were aged 7, 8, 10, and 12 years using items from the Social Behavior Questionnaire (SBQ^18^). The SBQ integrates items from the Rutter Children’s Behaviour Questionnaire,^19^ the Child Behavior Checklist,^20^ the Ontario Child Health Study scales,^21^ and the Preschool Behavior Questionnaire.^22^ Mothers and teachers ranked the frequency with which children experienced different dimensions of depression (eg, unhappy, sad, or depressed or lacked energy) on a scale from 0 (never) to 2 (often), with higher scores indicating more depression. Given our focus on depression symptoms, we used 5 SBQ items that were similar to the items used to assess depression in adolescence (ages 13-17 years) and young adulthood (ages 18-24 years) (eTable 1 in Supplement 1). The internal consistency of these items ranged from 0.19 (95% CI, 0.14-0.25) to 0.63 (95% CI, 0.60-0.65).

To create childhood depression variables, we first calculated the mean scores, separately, of mother-reported and teacher-reported depression symptoms. To account for variation in the measures used to assess depression symptoms across developmental periods, we identified children in the top quintile of mother-reported and teacher-reported depression symptom scores. These variables were used as binary indicators of early-childhood (mother-reported) and middle-childhood (teacher-reported) depression symptoms, in which 1 indicated children rated in the top quintile of depression symptoms by mothers and teachers, respectively, and 0 indicated all other children.

Adolescents self-reported their depression symptoms using the SBQ at age 13 years and at ages 15 and 17 years (α = 0.90), using the Mental Health and Social Inadaptation Assessment for Adolescents.^23^ We first calculated the mean of depression symptoms at ages 15 and 17 years using the Mental Health and Social Inadaptation Assessment for Adolescents and then identified participants in the top quintile of this mean score. We then identified participants in the top quintile of depression symptoms at age 13 years. The final variable was binary, with 1 indicating adolescents rated in the top quintile of depression symptoms at ages 13 or 15 and 17 years and 0 indicating all other children. To examine correlations between depression scores reported by different informants across different ages, we used the Spearman correlation coefficient. This test was used due to the nonnormal distribution of depression scores.

Outcomes in young adulthood were self-reported only at ages 20 and 21 years. At age 20 years, participants reported their depression symptoms using the Center for Epidemiologic Studies Depression (CES-D) scale,^24^ a validated and widely used measure of depression symptoms in adults. Psychosocial outcomes were reported at age 21 years. Perceived levels of stress in the past month were assessed using the Perceived Stress Scale.^25^ Social support was assessed using the validated short version of the Social Provisions Scale.^26^ Experiencing online harrasment was assessed using a single item asking about the frequency (never, once, sometimes, often, or very often) with which the participant had been harrased (eg, insults, threats) over the internet or by telephone in the past year. We created a binary variable with 1 for participants who indicated being harrased at least once and 0 otherwise. Binge drinking was also assessed with a single item asking how often participants had consumed 4 (for females) or 5 (for males) or more drinks on a single occasion in the past year. Participants’ NEET status was determined using 2 items asking about their current studies and employment. Participants who indicated that they were not in school, in training, or employed were classified as NEET.

### Covariates

We searched previous literature for variables that could confound associations between depression symptoms and each of the adult outcomes. Different covariates were used in different models, as each outcome was included in a separate model. All of the following covariates were assessed at baseline when children were aged 5 months old: family socioeconomic status (derived from parental educational and occupational status and household income), maternal and paternal depression symptoms (based on the CES-D scale^24^) and antisocial behavior in their adolescence and adulthood (assessed with 5 binary questions on conduct problems based on *Diagnostic and Statistical Manual of Mental Disorders* [Fourth Edition]^27^ criteria), maternal employment status, maternal substance use during pregnancy (ie, tobacco, alcohol, or an illegal drug), in-home observations of mother and child interactions (stimulation and verbalization) using the Home Observation Measurement of the Environment,^28^ self-reported maternal and paternal parenting practices (self-efficacy, reactive hostility, overprotection, affection, warmth, and parental impact) using the Parental Cognitions and Conduct Toward the Infant Scale,^29^ and the child’s sex. Family functioning was assessed using the Family Dysfunction Scale, in which scores range from 0 to 10.00, with higher scores indicating higher levels of family dysfunction.^30^

### Statistical Analysis

Data analysis was performed from October 4, 2022, to January 3, 2024. We estimated the association of early and middle childhood and adolescent depression symptoms with each adult outcome in separate regression models that were adjusted for the relevant covariates. Linear regression models were used for continuous outcomes (depression, perceived stress, and social support) and logistic regressions for binary outcomes (experiencing online harrasment, binge drinking, and NEET status). We also tested the interactions of depression symptoms in early childhood, middle childhood, and adolescence in each model. The interactions between depression symptoms in early childhood and at other time points were not significant and were therefore dropped from the models. Given the use of multiple testing, we present both the unadjusted and adjusted (Bonferroni-corrected) *P* values for all models; the Bonferroni correction was used for the final model of each outcome. A 2-sided *P* < .05 was considered significant.

Participants were included in analyses if they had available data for at least 1 time point for depression symptoms in early or middle childhood and adolescence and 1 adult outcome. The excluded and analytic samples significantly differed in baseline characteristics; we therefore used inverse probability weighting, in which weights represent the probability of being included in an analytic sample, in all analyses.^31^ The comparison of each analytic sample with the excluded sample on the variables used for weighting is presented in eTable 2 in Supplement 1. Missing data for covariates, ranging from 4.89% to 5.19% depending on the sample, were handled using multiple imputation by a chained equation (n = 50 imputed datasets). Statistical analyses were performed using R, version 4.2.3 (R Project for Statistical Computing).^32^

## Results

Among the 2120 infants in the cohort, the analysis sample size varied from 1118 to 1254 across outcomes and included 648 to 713 females (56.85% to 57.96%) and 470 to 541 males (42.04% to 43.14%) (Table 1). Participants who experienced high depression symptoms in adolescence were more likely to experience depression symptoms in young adulthood (β, 1.08 [95% CI, 0.84-1.32]; *P* < .001 unadjusted and Bonferroni adjusted) and to report higher levels of perceived stress (β, 3.63 [95% CI, 2.66-4.60]; *P* < .001 unadjusted and Bonferroni adjusted) after adjusting for covariates (Figure and eTable 3 in Supplement 1). Depression symptom scores were created in the cohort of 2120 infants, including the mean, SD, range, and cutoff scores for children and adolescents in the top quintile in each developmental period (early childhood: mean [SD] score, 1.18 [0.87; range, 0-7.14]; middle childhood: mean [SD] score, 1.88 [1.54; range, 0-10.00]; adolescence: mean [SD] score, 3.62 [2.08; range, 0-10.00]) (Table 2). The correlations between depression scores across different ages are presented in the eFigure in Supplement 1. Depression symptoms’ correlation coefficients were greater when reported by the same informant compared with coefficients between informants.

**Table 1.** Characteristics by Outcome^a^

Characteristic	Participants, No. (%) (N = 2120)^b^					
	Depression (n = 1177)^c^	Social support (n = 1254)^d^	Perceived stress (n = 1249)^e^	Experiencing online harrasment (n = 1254)^f^	Binge drinking (n = 1118)^g^	NEET (n = 1247)^h^
Sex						
Female	680 (57.77)	713 (56.86)	710 (56.85)	713 (56.86)	648 (57.96)	710 (56.94)
Male	497 (42.23)	541 (43.14)	539 (43.15)	541 (43.14)	470 (42.04)	537 (43.06)
Young adulthood outcome score, mean (SD)	2.51 (1.76)	26.52 (4.32)	17.67 (7.34)	NA	NA	NA
Young adulthood outcome						
No	NA	NA	NA	1026 (85.25)	583 (52.15)	1175 (94.23)
Yes	NA	NA	NA	185 (14.75)	535 (48.85)	72 (5.77)
Family socioeconomic status score, mean (SD)^i^	0.13 (0.97)	0.10 (0.98)	0.10 (0.98)	0.10 (0.98)	0.12 (0.99)	0.10 (0.98)
Maternal educational level						
No high school diploma	179 (15.23)	207 (16.53)	206 (16.52)	207 (16.53)	184 (16.49)	207 (16.63)
High school diploma	267 (22.72)	290 (23.16)	287 (23.02)	290 (23.16)	250 (22.40)	287 (23.05)
Post–high school diploma	359 (30.55)	368 (29.39)	367 (29.43)	368 (29.39)	322 (28.85)	364 (29.24)
University diploma	370 (31.49)	387 (30.91)	387 (31.03)	387 (30.91)	360 (32.26)	387 (31.08)
Parental age at child’s birth, mean (SD), y						
Mother	29.49 (5.04)	29.42 (5.13)	29.42 (5.12)	29.42 (5.13)	29.36 (5.10)	29.43 (5.12)
Father	32.26 (5.59)	32.24 (5.53)	32.24 (5.54)	32.24 (5.53)	32.18 (5.49)	32.25 (5.54)
Mother smoked during pregnancy						
No	884 (75.56)	946 (75.68)	942 (75.66)	946 (75.68)	941 (75.49)	939 (75.54)
Yes	286 (24.44)	304 (24.32)	303 (24.34)	304 (24.32)	273 (24.51)	304 (24.46)
Family functioning score, mean (SD)^j^	1.62 (1.39)	1.64 (1.41)	1.64 (1.41)	1.64 (1.41)	1.62 (1.13)	1.64 (1.41)
Family status						
Intact	978 (83.30)	1030 (82.27)	1027 (82.36)	1030 (82.27)	919 (82.35)	1025 (82.33)
Always single	127 (10.82)	139 (11.10)	138 (11.07)	139 (11.10)	124 (11.11)	138 (11.08)
Widowed	69 (5.88)	83 (6.63)	82 (6.58)	83 (6.63)	73 (6.54)	82 (6.59)
Positive mother and child interactions score, mean (SD)^k^	9.03 (1.04)	9.02 (1.05)	9.02 (1.04)	9.02 (1.05)	9.03 (1.03)	9.02 (1.05)
Parental depression score, mean (SD)^c^						
Mother	1.31 (1.27)	1.33 (1.28)	1.33 (1.28)	1.33 (1.28)	1.29 (1.26)	1.33 (1.28)
Father	0.97 (0.94)	1.01 (0.97)	1.01 (0.98)	1.01 (0.97)	1.00 (0.97)	1.01 (0.98)

Abbreviations: NA, not applicable; NEET, not in education, employment, or training.

^a^ Data were compiled from the final master file of the Québec Longitudinal Study of Child Development (from 1998 to 2019; Gouvernement du Québec, Institut de la Statistique du Québec).

^b^ Percentages are based on category response totals, which varied.

^c^ Based on the Center for Epidemiologic Studies Depression scale,^24^ in which scores range from 0 to 9.74, with higher scores indicating higher depression symptoms.

^d^ Based on the short version of the Social Provisions Scale,^26^ in which scores range from 0 to 30.00, with higher scores indicating higher perceived social support.

^e^ Based on the Perceived Stress Scale,^25^ in which scores range from 0 to 40.00, with higher scores indicating higher perceived stress.

^f^ Victimization frequency over the internet or by telephone in the past year.

^g^ Consumption frequency of 4 (females) or 5 (males) or more drinks on a single occasion in the past year.

^h^ Neither in school nor employed.

^i^ Derived from parental educational and occupational status and household income. Scores range from −3.01 to 2.85, with higher scores indicating higher levels of socioeconomic status.

^j^ Self-reported family functioning using the Family Dysfunction Scale, in which scores range from 0 to 10.00, with higher scores indicating higher levels of family dysfunction.^30^

^k^ In-home observations of mother and child interactions (stimulation and verbalization) using the Home Observation Measurement of the Environment,^28^ in which scores range from 3.50 to 10.00, with higher scores indicating more frequent mother-child interactions.

**Figure.**
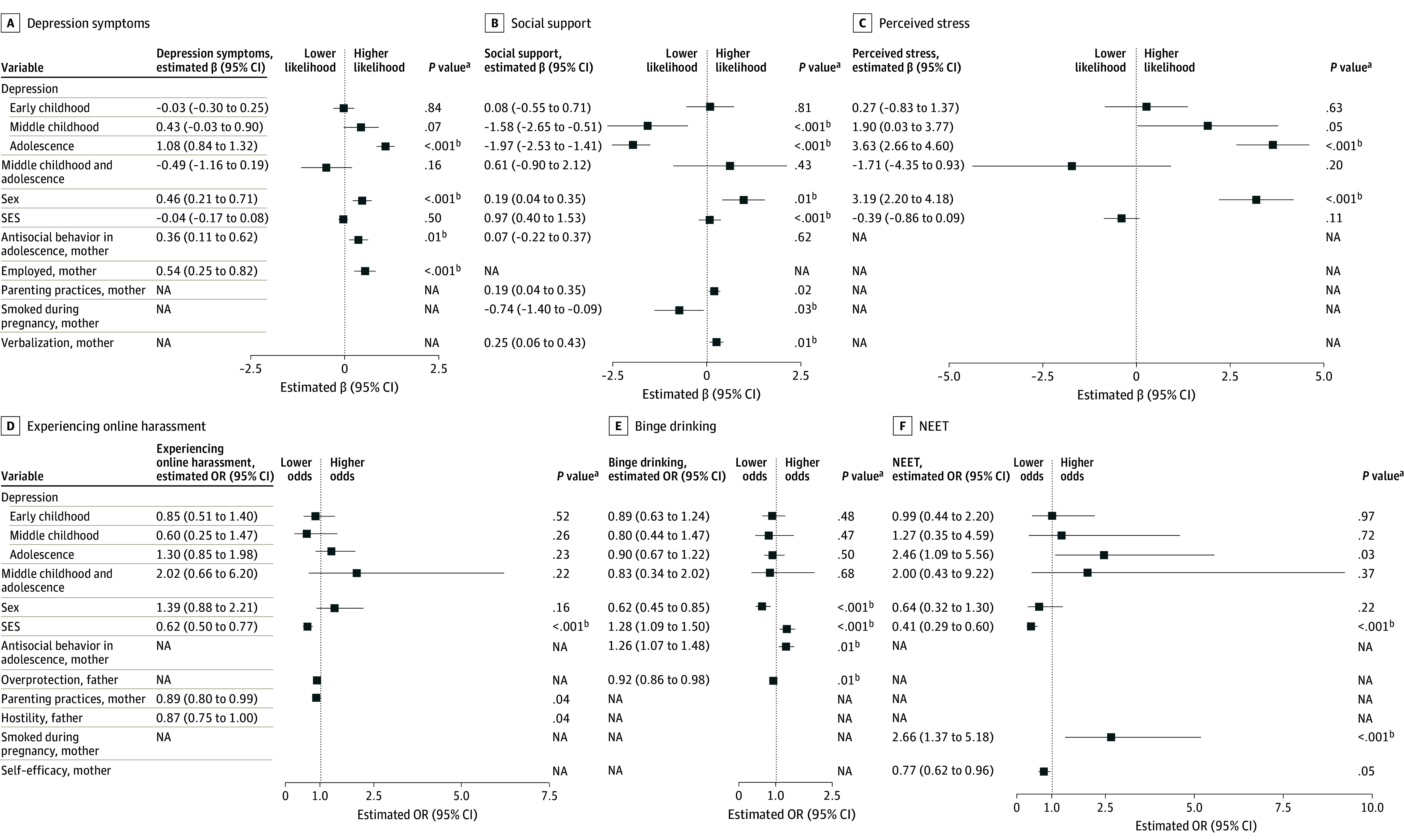
Associations of Early- and Middle-Childhood and Adolescent Depression With Outcomes in Young Adulthood Data were compiled from the final master file of the Québec Longitudinal Study of Child Development (1998–2019), Gouvernement du Québec, Institut de la Statistique du Québec (Quebec Institute of Statistics). Details on the scales used and scoring are found in the Methods section and Table 1. NEET indicates not being in education, employment, or training; OR, odds ratio; and SES, socioeconomic status. ^a^NA (not applicable) represents variables that were not kept in the final model because they did not reach statistical significance. ^b^Factors remaining significant (*P* < .05) after applying Bonferroni adjustment.

**Table 2.** Depression Symptom Scores Across Developmental Periods^a^

Depression symptom period	Score, mean (SD) [range]	Score top quintile
Early childhood^b^	1.18 (0.87) [0-7.14]	2.00
Middle childhood^b^	1.88 (1.54) [0-10.00]	3.33
Adolescence^c^	3.62 (2.08) [0-10.00]	5.32

^a^ Data were compiled from the final master file of the Québec Longitudinal Study of Child Development (from 1998 to 2019), the Gouvernement du Québec, and the Institut de la Statistique du Québec.

^b^ Overall score of the parent (early childhood; ages 1.5 to 6 years) and teacher (middle childhood; ages 7 to 12 years) responses to the Social Behavior Questionnaire (SBQ)^18^ regarding the frequency with which children experienced different dimensions of depression on a scale from 0 (never) to 2 (often) for individual items, with higher scores indicating more depression.

^c^ Self-reported symptoms using the SBQ^18^ (age 13 years) and the Mental Health Social Inadaptation Assessment for Adolescents^23^ (ages 15 and 17 years), in which scores range from 0 to 10.00, with higher scores indicating higher depression symptoms.

High depression symptoms in middle childhood were not associated with higher levels of depression symptoms (β, 0.43 [95% CI, −0.03 to 0.90]; *P* = .07) and perceived stress (β, 1.90 [95% CI, 0.03-3.77]; *P* = .05) in young adulthood; these results remained nonsignificant after adjusting for multiple testing (depression symptoms: β, 0.43 [95% CI, −0.03 to 0.90]; *P* = .11 and perceived stress: β, 1.90 [95% CI, 0.03-3.77]; *P* = .10) (eTable 3 in Supplement 1). A similar pattern was observed between high depression symptoms in adolescence and NEET status in young adulthood, in which the statistical significance (β, 2.46 [95% CI, 1.09-5.56]; *P* = .03) did not survive the Bonferroni correction (*P* = .06) (eTable 3 in Supplement 1).

The only outcome with which high depression symptoms in middle childhood and adolescence were associated was social support (Figure and eTable 3 in Supplement 1). Participants in middle childhood (β, −1.58 [95% CI, −2.65 to −0.51]; *P* = .003 unadjusted and *P* < .001 Bonferroni adjusted) and adolescents (β, −1.97 [95% CI, −2.53 to −1.41]; *P* < .001 unadjusted and Bonferroni adjusted) who experienced more depression symptoms reported lower levels of social support in young adulthood. The interaction between high depression symptoms in middle childhood and adolescence was not significant, suggesting that the independent associations of depression symptoms in each period were more relevant than the cumulative experience of high depression symptoms (Figure and eTable 3 in Supplement 1). We found no association between high depression symptoms across developmental periods with any outcome. The experience of high depression symptoms across (early and middle) childhood and adolescence was not associated with binge drinking, NEET status, or experiencing online harrasment (Figure and eTable 3 in Supplement 1). To test whether the associations of depression symptoms with adult outcomes were mediated by depression symptoms at a later point, we conducted simple regression analyses between depression symptoms in each developmental period and adult outcomes to ensure that the impact of childhood depression symptoms was not overshadowed by later depression symptoms (eTable 4 in Supplement 1). As almost all of the results were not significant, it appeared that the association of childhood depression symptoms with adult outcomes was not masked by later depression symptoms, and therefore, we did not test mediation models.

## Discussion

In this cohort study using prospective longitudinal data from children, adolescents, and adults aged 1.5 to 21 years, we found that depression symptoms during adolescence were associated with increased depression symptoms at age 20 years and perceived stress at age 21 years, adjusting for covariates and multiple testing. Additionally, depression symptoms during adolescence were associated with compromised psychosocial outcomes at age 21 years, but the result was nonsignificant after correcting for multiple testing. Social support was the only outcome for which depression symptoms during middle childhood and adolescence had an association that persisted after adjusting for covariates and multiple testing. Depression symptoms were not associated with experiencing online harrasment, NEET status, or binge drinking.

Being in the top quintile of depression symptoms in adolescence was associated with a 1-point increase on the CES-D scale, an association that corresponds with a medium effect size (Cohen *d* = 0.5) and is thus relevant from a population and clinical perspective. These findings provide some support for the stability of depression symptoms and are consistent with previous research suggesting that depression symptoms during adolescence increase the risk of mental health problems in emerging adulthood.^7^ Additionally, in our study, young adults who experienced depression symptoms during adolescence self-reported increased perceived stress at age 21 years, independent of early risk factors. One explanation for this finding is that the experience of depression symptoms may have contributed to cognitive vulnerabilities and the perception of events as more stressful. Alternatively, it could be that young adults may have experienced stressful life circumstances at the time of the assessment or that structural or social determinants not captured at birth may have contributed to depression symptoms.^33,34,35^ Notably, these results were not significant for childhood depression symptoms, suggesting that the associations were confined to adolescent depression symptoms. However, it is worth mentioning that adolescence and adult depression symptoms were measured with self-reports, which may have reflected common rater bias, while early- and middle-childhood depression symptoms were measured with mothers’ (early childhood) and teachers’ (middle childhood) reports, which may have reflected measurement and rater difference.^36^

The experience of depression symptoms in middle childhood and adolescence was associated with decreased social support at age 21 years. There were no significant interactions, suggesting that the independent associations of depression symptoms in each developmental period were more relevant than the cumulative experience of high depression symptoms. This finding is consistent with a previous study’s finding that adolescent depression symptoms were associated with lower social support in early adulthood^37^ and adds to the existing literature by showing that the experience of depression symptoms during middle childhood (ages 7 to 12 years) may be independently associated with diminished social support. This is a concerning finding, as it implies that young adults may go through life transitions (eg, family and career) without adequate social support.^38^ Similarly, they may be reluctant to access support provided by health services.^37,38^ Future research should examine why this occurs and if the associations of childhood vs adolescent depression with social support have distinct environmental and genetic causes.

While no firm conclusions can be made about the timing (childhood vs adolescence) of depression symptoms and its prospective associations with adult outcomes, it appears that depression symptoms during adolescence were associated with a broader range of adult outcomes (depression symptoms, perceived stress, and social support) compared with depression symptoms during childhood (social support only). There was no evidence that individuals with persistently elevated depression symptoms relative to peers had worse adult outcomes. Except for social support, young adults whose depression symptoms did not persist beyond childhood showed no other impairments, suggesting that it was depression symptoms in adolescence that were associated with adult outcomes. However, this finding should be interpreted with caution because the association may be an artifact of the fact that depression symptoms were reported by different informants at different ages with different measures.^36^

### Limitations

The onset and course of depression symptoms were not captured in this study. Future studies should examine trajectories of depression symptoms and their prospective associations with adult outcomes. Moreover, the overall low internal consistency of depression items in early childhood, reported by mothers and teachers, has to be considered, as it indicates a potential lack of validity of depression measures. There were no data on whether participants were treated with antidepressant medication or psychological therapy, which may have impacted depression symptoms and adult outcomes. Exposure variables during adolescence and outcomes in early adulthood were assessed using self-reports, which may have inflated associations between variables (eg, individuals experiencing depression being more vulnerable to negative self-perceptions).^39,40^ However, different reporters (mothers, teachers) were used to measure depression symptoms across childhood, and self-reports are reliable for internalizing problems.^41,42^

The findings have implications for mental health interventions. It is of clinical importance to identify children and adolescents experiencing depression early to decrease depression symptoms and prevent compromised functioning. Our findings suggest that mental health interventions including interpersonal/social components may improve psychosocial functioning in adulthood. Furthermore, some of the early risk factors we considered showed associations with adverse adult outcomes. Thus, mental health interventions that address exposure to early adversity or trauma could be beneficial to children and adolescents experiencing depression symptoms.^43^ Last, mental health interventions should identify and monitor children and adolescents experiencing subclinical symptoms as our findings suggest that individuals who had increased depression symptoms during childhood or adolescence experienced adverse outcomes in young adulthood.

## Conclusions

The findings of this cohort study suggest that both childhood and adolescent depression symptoms may be associated with adverse psychosocial outcomes, while adolescent depression symptoms were associated with depression symptoms and perceived stress in young adulthood independent of early risk factors. Interventions should aim to screen and monitor children and adolescents for depression to inform policymaking regarding young adult mental health and psychosocial outcomes.

## Notes

Supplement 1. **eTable 1.** MIA and SBQ Depression Symptoms Items **eFigure.** Correlation Plot of All Depression Symptoms Scores **eTable 2.** Comparison of Included and Excluded Participants for Each Outcome **eTable 3.** Estimated Coefficients (β or OR) Associated With an Increased Risk of Reporting Impaired Adult Outcomes **eTable 4.** Estimated Coefficients (β or OR) of Unadjusted and Adjusted Depression Symptoms at Every Time Point Associated With an Increased Risk of Reporting Impaired Adult Outcomes

Supplement 2.
Data Sharing Statement
