# Supplementary material for: Childhood and Adolescent Depression Symptoms and Young Adult Mental Health and Psychosocial Outcomes
Source: JAMA Netw Open. 2024 Aug 8;7(8):e2425987. doi: 10.1001/jamanetworkopen.2024.25987 (PMC11310820; doi:10.1001/jamanetworkopen.2024.25987)
Supplement: Supplement 1. — eTable 1. MIA and SBQ Depression Symptoms Items eFigure. Correlation Plot of All Depression Symptoms Scores eTable 2. Comparison of Included and Excluded Participants for Each Outcome eTable 3. Estimated Coefficients (β or OR) Associated With an Increased Risk of Reporting Impaired Adult Outcomes eTable 4. Estimated Coefficients (β or OR) of Unadjusted and Adjusted Depression Symptoms at Every Time Point Associated With an Increased Risk of Reporting Impaired Adult Outcomes [file jamanetwopen-e2425987-s001.pdf]

Supplemental Online Content

Psychogiou L, Navarro MC, Orri M, Côté SM, Ahun MN. Childhood and adolescent depression symptoms and young adult mental health and psychosocial outcomes. *JAMA Netw Open*. 2024;7(8):e2425987. doi:10.1001/jamanetworkopen.2024.25987

eTable 1. MIA and SBQ Depression Symptoms Items

eFigure. Correlation Plot of All Depression Symptoms Scores

eTable 2. Comparison of Included and Excluded Participants for Each Outcome

eTable 3. Estimated Coefficients (β or OR) Associated With an Increased Risk of Reporting Impaired Adult Outcomes

eTable 4. Estimated Coefficients (β or OR) of Unadjusted and Adjusted Depression Symptoms at Every Time Point Associated With an Increased Risk of Reporting Impaired Adult Outcomes

This supplemental material has been provided by the authors to give readers additional information about their work.

© 2024 Psychogiou L et al. *JAMA Network Open*.

eTable 1. MIA and SBQ Depression Symptoms Items

MIA depression symptoms items	SBQ depression symptoms items
I felt sad and unhappy	Seemed to be unhappy or sad
I felt I wasn't as good-looking or as smart as other people	Was not as happy as other children
I felt I couldn't do anything well	
Nothing was fun for me, I wasn't interested in anything	Had trouble enjoying him/herself
I lost interest in things I usually like	
I lacked energy or felt tired	Had no energy, was feeling tired
Doing even little things made me feel really tired	
I had trouble thinking clearly	Is incapable of making decisions

© 2024 Psychogiou L et al. *JAMA Network Open*.

eFigure. Correlation Plot of All Depression Symptoms Scores

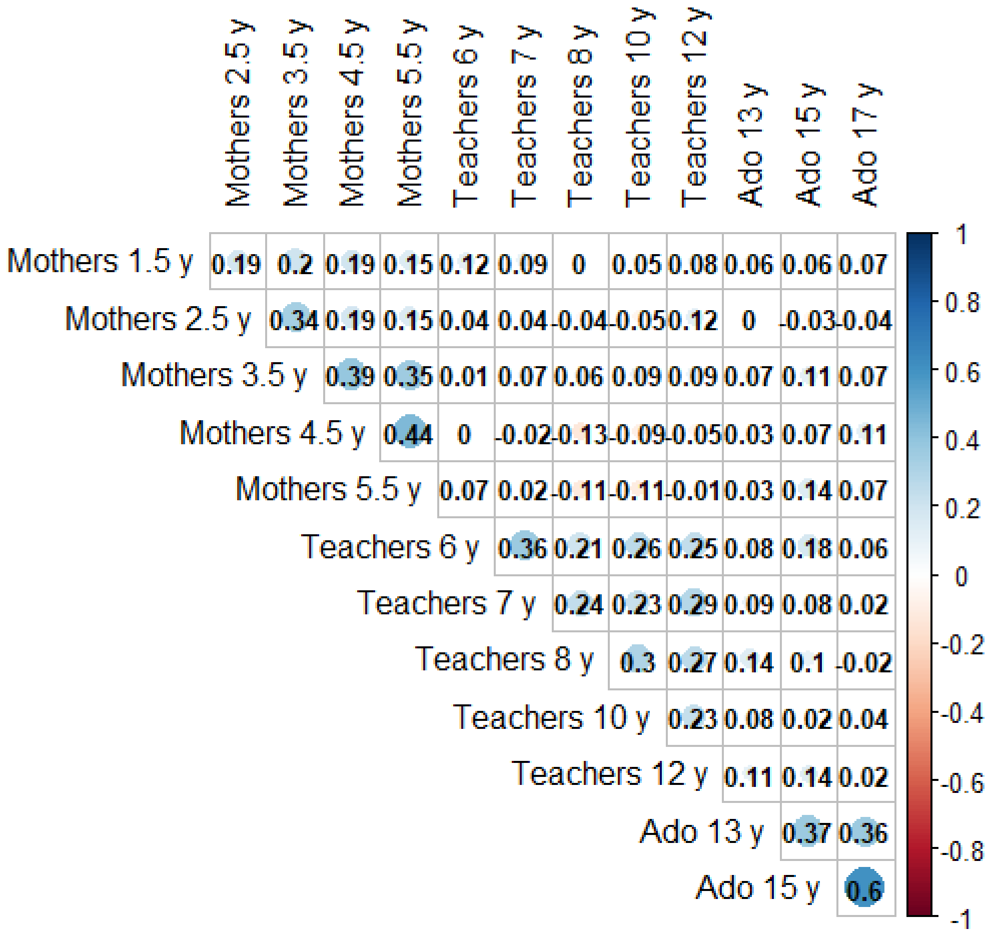

© 2024 Psychogiou L et al. *JAMA Network Open*.

eTable 2. Comparison of Included and Excluded Participants for Each Outcome

	**Depression symptoms**			**Social Support & Cybervictimization**			**Perceived stress**		
	Included (n=1,177)	Excluded (n=943)	p	Included (n=1,254)	Excluded (n=866)	p	Included (n=1,249)	Excluded (n=871)	p
**Sex, n (%)**			<0.01			<0.01			<0.01
Boys	497 (42.23)	524 (63.13)		541 (43.14)	480 (63.75)		539 (43.15)	482 (63.59)	
Girls	680 (57.77)	306 (36.87)		713 (56.86)	273 (36.25)		710 (56.85)	276 (36.41)	
**Socioeconomic status, mean (SD)**	0.13 (0.97)	-0.20 (1.00)	<0.01	0.10 (0.98)	-0.18 (1.00)	<0.01	0.10 (0.98)	-0.18 (1.00)	<0.01
**Maternal education, n (%)**			<0.01			<0.01			<0.01
No high school diploma	179 (15.23)	184 (22.20)		207 (16.53)	156 (20.74)		206 (16.52)	157 (20.74)	
High school diploma	267 (22.72)	254 (30.64)		290 (23.16)	231 (30.72)		287 (23.02)	234 (30.91)	
Post high school diploma	359 (30.55)	221 (26.66)		368 (29.39)	212 (28.19)		367 (29.43)	213 (28.14)	
University diploma	370 (31.49)	170 (20.51)		387 (30.91)	153 (20.35)		387 (31.03)	153 (20.21)	
**Father highest diploma, n (%)**			<0.01			<0.01			<0.01
No high school diploma	206 (18.73)	177 (24.25)		222 (19.06)	161 (24.21)		221 (19.04)	162 (24.22)	
High school diploma	256 (23.27)	210 (28.77)		284 (24.38)	182 (27.37)		282 (24.29)	184 (27.50)	
Post high school diploma	324 (29.45)	198 (27.12)		340 (29.18)	182 (27.37)		339 (29.20)	183 (27.35)	
University diploma	314 (28.55)	145 (19.86)		319 (27.38)	140 (21.05)		319 (27.48)	140 (20.93)	
**Family type, n (%)**			<0.01			<0.01			<0.01
Intact	978 (83.30)	638 (77.15)		1,030 (82.27)	586 (78.24)		1,027 (82.36)	589 (78.12)	
Always single	127 (10.82)	96 (11.61)		139 (11.10)	84 (11.21)		138 (11.07)	85 (11.27)	
Widowed	69 (5.88)	93 (11.25)		83 (6.63)	79 (10.55)		82 (6.58)	80 (10.61)	
**Mother age at birth, mean (SD)**	29.49 (5.04)	28.93 (5.50)	0.01	29.42 (5.13)	29 (5.43)	0.07	29.42 (5.12)	29 (5.43)	0.06
**Father age at birth, mean (SD)**	32.26 (5.59)	32.12 (5.83)	0.5	32.24 (5.53)	32.13 (5.94)	0.5	32.24 (5.54)	32.13 (5.93)	0.5
**Child birth rank, n (%)**			0.3			0.06			0.07
1	540 (45.88)	350 (42.17)		573 (45.69)	317 (42.10)		571 (45.72)	319 (42.08)	
2	457 (38.83)	343 (41.33)		495 (39.47)	305 (40.50)		493 (39.47)	307 (40.50)	
3	129 (10.96)	89 (10.72)		137 (10.93)	81 (10.76)		136 (10.89)	82 (10.82)	
4	33 (2.80)	29 (3.49)		32 (2.55)	30 (3.98)		32 (2.56)	30 (3.96)	
5	18 (1.53)	19 (2.29)		17 (1.36)	20 (2.66)		17 (1.36)	20 (2.64)	
**Maternal consumption during pregnancy, n (%)**									
**Smoke**			0.2			0.14			0.15
No	884 (75.56)	605 (73.16)		946 (75.68)	543 (72.69)		942 (75.66)	547 (72.74)	
Yes	286 (24.44)	222 (26.84)		304 (24.32)	204 (27.31)		303 (24.34)	205 (27.26)	
**Alcohol (number of glass)**			<0.01			<0.01			<0.01
Never	702 (60)	572 (69.17)		755 (60.40)	519 (69.48)		751 (60.32)	523 (69.55)	
Less than one time per month	353 (30.17)	193 (23.34)		379 (30.32)	167 (22.36)		379 (30.44)	167 (22.21)	
Once per week	65 (5.56)	44 (5.32)		66 (5.28)	43 (5.76)		65 (5.22)	44 (5.85)	

© 2024 Psychogiou L et al. *JAMA Network Open*.

More than once per week	50 (4.27)	18 (2.18)		50 (4)	18 (2.41)		50 (4.02)	18 (2.39)	
**Illegal drugs**			0.4			0.7			0.7
No	1,155 (98.72)	813 (98.31)		1,231 (98.48)	737 (98.66)		1,226 (98.47)	742 (98.67)	
Yes	15 (1.28)	14 (1.69)		19 (1.52)	10 (1.34)		19 (1.53)	10 (1.33)	
**Maternal mental health, mean (SD)**									
Depression	1.31 (1.27)	1.54 (1.42)	<0.01	1.33 (1.28)	1.52 (1.42)	0.01	1.33 (1.28)	1.52 (1.43)	0.01
Antisocial behavior in adolescence	0.81 (0.93)	0.84 (0.97)	0.6	0.81 (0.92)	0.85 (0.99)	0.8	0.81 (0.92)	0.84 (0.99)	0.8
Antisocial behavior in adulthood	0.21 (0.46)	0.23 (0.54)	0.7	0.20 (0.45)	0.24 (0.56)	0.4	0.20 (0.45)	0.24 (0.56)	0.4
**Paternal mental health, mean (SD)**									
Depression	0.97 (0.94)	1.06 (1.03)	0.12	1.01 (0.97)	1 (0.97)	>0.9	1.01 (0.98)	1 (0.97)	0.9
Antisocial behavior in adolescence	0.65 (0.93)	0.72 (1.01)	0.4	0.66 (0.92)	0.71 (1.02)	0.8	0.66 (0.93)	0.71 (1.02)	0.8
Antisocial behavior in adulthood	0.57 (0.79)	0.53 (0.80)	0.2	0.56 (0.79)	0.54 (0.81)	0.5	0.56 (0.79)	0.54 (0.81)	0.5
**Mother-Child interactions, mean (SD)**									
Positive interactions	9.03 (1.04)	9.01 (1.08)	0.8	9.02 (1.05)	9.02 (1.07)	0.9	9.02 (1.04)	9.01 (1.08)	>0.9
Verbalization	6.75 (1.60)	6.51 (1.69)	<0.01	6.76 (1.59)	6.48 (1.72)	<0.01	6.76 (1.59)	6.48 (1.72)	<0.01
Stimulation	4.82 (2.37)	4.56 (2.36)	0.01	4.82 (2.38)	4.54 (2.34)	0.02	4.83 (2.38)	4.53 (2.34)	0.01
**Family functioning, mean (SD)**	1.62 (1.39)	1.80 (1.54)	0.03	1.64 (1.41)	1.78 (1.52)	0.08	1.64 (1.41)	1.78 (1.52)	0.09
**Maternal parenting practices, mean (SD)**									
Perceived efficacy	8.79 (1.12)	8.78 (1.17)	0.6	8.78 (1.12)	8.80 (1.17)	0.3	8.78 (1.13)	8.80 (1.17)	0.2
Perceived impact	8.47 (1.79)	8.25 (1.98)	0.06	8.46 (1.77)	8.23 (2.03)	0.2	8.47 (1.76)	8.22 (2.05)	0.10
Perceived reactive hostility	1.07 (1.46)	1.05 (1.42)	0.7	1.07 (1.44)	1.05 (1.46)	0.2	1.07 (1.43)	1.05 (1.46)	0.2
Perceived affection	9.69 (0.72)	9.69 (0.68)	0.3	9.68 (0.70)	9.69 (0.70)	0.12	9.68 (0.71)	9.69 (0.70)	0.11
Perceived overprotection	5.24 (2.42)	5.58 (2.39)	<0.01	5.28 (2.40)	5.56 (2.42)	<0.01	5.27 (2.40)	5.57 (2.42)	<0.01
Awareness of child's qualities	7.95 (1.73)	7.87 (1.79)	0.5	7.95 (1.72)	7.87 (1.81)	0.6	7.95 (1.71)	7.87 (1.82)	0.6
**Paternal parenting practices, mean (SD)**									
Perceived efficacy	7.72 (1.63)	7.82 (1.63)	0.2	7.67 (1.66)	7.92 (1.56)	<0.01	7.67 (1.67)	7.92 (1.55)	<0.01
Perceived impact	8.46 (1.77)	8.23 (1.84)	<0.01	8.45 (1.75)	8.23 (1.89)	0.07	8.45 (1.75)	8.23 (1.89)	0.05
Perceived reactive hostility	1.31 (1.60)	1.30 (1.65)	0.6	1.32 (1.60)	1.28 (1.66)	0.2	1.32 (1.60)	1.27 (1.66)	0.2
Perceived affection	9.07 (1.18)	9 (1.28)	0.6	9.03 (1.20)	9.08 (1.25)	0.07	9.03 (1.20)	9.08 (1.25)	0.07
Perceived overprotection	4.01 (2.35)	4.30 (2.36)	0.02	3.98 (2.34)	4.37 (2.38)	<0.01	3.98 (2.34)	4.38 (2.38)	<0.01
Awareness of child's qualities	7.86 (1.66)	7.87 (1.69)	0.9	7.86 (1.66)	7.89 (1.69)	0.6	7.86 (1.66)	7.89 (1.69)	0.6

© 2024 Psychogiou L et al. *JAMA Network Open*.

**Mother worked at survey, n (%)**			0.6			>0.9			>0.9
No	946 (81.69)	675 (82.62)		1,012 (82.08)	609 (82.08)		1,007 (82)	614 (82.20)	
Yes	212 (18.31)	142 (17.38)		221 (17.92)	133 (17.92)		221 (18)	133 (17.80)	
**Mother working status past 12 months, n (%)**			0.01			0.2			0.12
Full-time job	634 (54.28)	413 (50.49)		671 (53.90)	376 (50.74)		669 (53.95)	378 (50.67)	
Part-time job	212 (18.15)	129 (15.77)		218 (17.51)	123 (16.60)		218 (17.58)	123 (16.49)	
No job	322 (27.57)	276 (33.74)		356 (28.59)	242 (32.66)		353 (28.47)	245 (32.84)	
**Child difficult temperament, mean (SD)**	2.67 (1.59)	2.75 (1.64)	0.3	2.68 (1.61)	2.74 (1.61)	0.5	2.68 (1.61)	2.74 (1.61)	0.4

© 2024 Psychogiou L et al. *JAMA Network Open*.

	Binge drinking			NEET		
	Included (n=1,118)	Excluded (n=1,002)	p	Included (n=1,247)	Excluded (n=873)	p
**Sex, n (%)**			<0.01			<0.01
Boys	470 (42.04)	551 (61.98)		537 (43.06)	484 (63.68)	
Girls	648 (57.96)	338 (38.02)		710 (56.94)	276 (36.32)	
**Socioeconomic status, mean (SD)**	0.12 (0.99)	-0.17 (0.99)	<0.01	0.10 (0.98)	-0.18 (0.99)	<0.01
**Maternal education, n (%)**			<0.01			<0.01
No high school diploma	184 (16.49)	179 (20.16)		207 (16.63)	156 (20.55)	
High school diploma	250 (22.40)	271 (30.52)		287 (23.05)	234 (30.83)	
Post high school diploma	322 (28.85)	258 (29.05)		364 (29.24)	216 (28.46)	
University diploma	360 (32.26)	180 (20.27)		387 (31.08)	153 (20.16)	
**Father highest diploma, n (%)**			<0.01			<0.01
No high school diploma	192 (18.48)	191 (24.15)		221 (19.07)	162 (24.14)	
High school diploma	247 (23.77)	219 (27.69)		282 (24.33)	184 (27.42)	
Post high school diploma	310 (29.84)	212 (26.80)		337 (29.08)	185 (27.57)	
University diploma	290 (27.91)	169 (21.37)		319 (27.52)	140 (20.86)	
**Family type, n (%)**			0.02			<0.01
Intact	919 (82.35)	697 (78.76)		1,025 (82.33)	591 (78.17)	
Always single	124 (11.11)	99 (11.19)		138 (11.08)	85 (11.24)	
Widowed	73 (6.54)	89 (10.06)		82 (6.59)	80 (10.58)	
**Mother age at birth, mean (SD)**	29.36 (5.10)	29.13 (5.42)	0.2	29.43 (5.12)	28.99 (5.43)	0.05
**Father age at birth, mean (SD)**	32.18 (5.49)	32.24 (5.93)	0.9	32.25 (5.54)	32.11 (5.93)	0.4
**Child birth rank, n (%)**			<0.01			0.05
1	518 (46.33)	372 (41.84)		570 (45.71)	320 (42.11)	
2	443 (39.62)	357 (40.16)		492 (39.45)	308 (40.53)	
3	118 (10.55)	100 (11.25)		137 (10.99)	81 (10.66)	
4	26 (2.33)	36 (4.05)		31 (2.49)	31 (4.08)	
5	13 (1.16)	24 (2.70)		17 (1.36)	20 (2.63)	
**Maternal consumption during pregnancy, n (%)**						
**Smoke**			0.3			0.2
No	841 (75.49)	648 (73.39)		939 (75.54)	550 (72.94)	
Yes	273 (24.51)	235 (26.61)		304 (24.46)	204 (27.06)	
**Alcohol (Frequency)**			<0.01			<0.01
Never	664 (59.61)	610 (69.08)		750 (60.34)	524 (69.50)	
Less than one time per month	345 (30.97)	201 (22.76)		379 (30.49)	167 (22.15)	
Once per week	58 (5.21)	51 (5.78)		64 (5.15)	45 (5.97)	
More than once per week	47 (4.22)	21 (2.38)		50 (4.02)	18 (2.39)	
**Illegal drugs**			0.3			0.7

© 2024 Psychogiou L et al. *JAMA Network Open*.

No	1,095 (98.29)	873 (98.87)		1,224 (98.47)	744 (98.67)	
Yes	19 (1.71)	10 (1.13)		19 (1.53)	10 (1.33)	
**Maternal mental health, mean (SD)**						
Depression	1.29 (1.26)	1.54 (1.42)	<0.01	1.33 (1.28)	1.52 (1.42)	<0.01
Antisocial behavior in adolescence	0.81 (0.93)	0.84 (0.97)	0.8	0.81 (0.92)	0.85 (0.99)	0.7
Antisocial behavior in adulthood	0.20 (0.46)	0.23 (0.54)	>0.9	0.20 (0.45)	0.24 (0.56)	0.4
**Paternal mental health, mean (SD)**						
Depression	1 (0.97)	1.01 (0.98)	0.8	1.01 (0.98)	1.00 (0.97)	0.9
Antisocial behavior in adolescence	0.66 (0.92)	0.70 (1.01)	0.8	0.66 (0.92)	0.71 (1.03)	0.8
Antisocial behavior in adulthood	0.56 (0.79)	0.55 (0.81)	0.6	0.56 (0.79)	0.55 (0.81)	0.7
**Mother-Child interactions, mean (SD)**						
Positive interactions	9.03 (1.03)	9.01 (1.08)	>0.9	9.02 (1.05)	9.02 (1.07)	>0.9
Verbalization	6.79 (1.58)	6.47 (1.71)	<0.01	6.76 (1.59)	6.48 (1.72)	<0.01
Stimulation	4.86 (2.40)	4.53 (2.32)	<0.01	4.83 (2.38)	4.53 (2.35)	0.01
**Family functioning, mean (SD)**	1.62 (1.43)	1.78 (1.48)	0.02	1.64 (1.41)	1.78 (1.51)	0.08
**Maternal parenting practices, mean (SD)**						
Perceived efficacy	8.79 (1.08)	8.78 (1.22)	0.3	8.78 (1.13)	8.80 (1.17)	0.3
Perceived impact	8.51 (1.71)	8.21 (2.05)	0.03	8.47 (1.76)	8.22 (2.04)	0.12
Perceived reactive hostility	1.08 (1.44)	1.05 (1.45)	0.2	1.07 (1.44)	1.05 (1.46)	0.2
Perceived affection	9.70 (0.60)	9.66 (0.81)	0.3	9.68 (0.71)	9.69 (0.70)	0.12
Perceived overprotection	5.24 (2.39)	5.56 (2.43)	<0.01	5.27 (2.40)	5.56 (2.43)	<0.01
Awareness of child's qualities	7.97 (1.67)	7.85 (1.85)	0.6	7.95 (1.72)	7.87 (1.81)	0.6
**Paternal parenting practices, mean (SD)**						
Perceived efficacy	7.68 (1.65)	7.87 (1.60)	0.01	7.68 (1.65)	7.90 (1.59)	<0.01
Perceived impact	8.49 (1.74)	8.21 (1.88)	<0.01	8.45 (1.75)	8.24 (1.88)	0.08
Perceived reactive hostility	1.35 (1.62)	1.23 (1.62)	0.06	1.32 (1.60)	1.27 (1.66)	0.2
Perceived affection	9.03 (1.21)	9.07 (1.22)	0.2	9.03 (1.20)	9.07 (1.25)	0.11
Perceived overprotection	3.93 (2.32)	4.38 (2.39)	<0.01	3.99 (2.33)	4.36 (2.39)	<0.01
Awareness of child's qualities	7.87 (1.65)	7.87 (1.69)	0.8	7.86 (1.66)	7.88 (1.69)	0.6
**Mother worked at survey, n (%)**			>0.9			0.9
No	903 (82.09)	718 (82.06)		1,005 (81.97)	616 (82.24)	
Yes	197 (17.91)	157 (17.94)		221 (18.03)	133 (17.76)	
**Mother working status past 12 months, n (%)**			<0.01			0.11
Full-time job	608 (54.82)	439 (50.06)		668 (53.96)	379 (50.67)	
Part-time job	199 (17.94)	142 (16.19)		218 (17.61)	123 (16.44)	
No job	302 (27.23)	296 (33.75)		352 (28.43)	246 (32.89)	
**Child difficult temperament, mean (SD)**	2.70 (1.59)	2.71 (1.64)	>0.9	2.69 (1.61)	2.73 (1.61)	0.5

Samples, for each outcome, were weighted for every variable whose p-value was lower than 0.05 when comparing included and excluded participants.

© 2024 Psychogiou L et al. *JAMA Network Open*.

eTable 3. Estimated Coefficients (β or OR) Associated With an Increased Risk of Reporting Impaired Adult Outcomes

	Depression symptoms			Social support			Perceived stress		
	**β**	**95% CI**	**p-value**	**β**	**95% CI**	**p-value**	**β**	**95% CI**	**p-value**
Early depression	-0.03	(-0.30, 0.25)	0.84	0.08	(-0.55, 0.71)	0.81	0.27	(-0.83, 1.37)	0.63
Middle depression	0.43	(-0.03, 0.90)	0.07	-1.58	(-2.65, -0.51)	0.00*	1.90	(0.03, 3.77)	0.05
Ado depression	1.08	(0.84, 1.32)	0.00*	-1.97	(-2.53, -1.41)	0.00*	3.63	(2.66, 4.60)	0.00*
Middle x Ado depression	-0.49	(-1.16, 0.19)	0.16	0.61	(-0.90, 2.12)	0.43	-1.71	(-4.35, 0.93)	0.20
Sex	0.46	(0.21, 0.71)	0.00*	0.19	(0.04, 0.35)	0.01*	3.19	(2.20, 4.18)	0.00*
SES	-0.04	(-0.17, 0.08)	0.50	0.97	(0.40, 1.53)	0.00*	-0.39	(-0.86, 0.09)	0.11
Mother adult. antisocial	0.36	(0.11, 0.62)	0.01*	0.07	(-0.22, 0.37)	0.62	-	-	-
Mother worked at the survey	0.54	(0.25, 0.82)	0.00*	-	-	-	-	-	-
Mother perceived child qualities				0.19	(0.04, 0.35)	0.02*			
Smoke pregnancy	-	-	-	-0.74	(-1.40, -0.09)	0.03*	-	-	-
Verbalization	-	-	-	0.25	(0.06, 0.43)	0.01*	-	-	-

	Cybervictimization			Binge drinking			NEET		
	**OR**	**95% CI**	**p-value**	**OR**	**95% CI**	**p-value**	**OR**	**95% CI**	**p-value**
Early depression	0.85	(0.51, 1.40)	0.52	0.89	(0.63, 1.24)	0.48	0.99	(0.44, 2.20)	0.97
Middle depression	0.60	(0.25, 1.47)	0.26	0.80	(0.44, 1.47)	0.47	1.27	(0.35, 4.59)	0.72
Ado depression	1.30	(0.85, 1.98)	0.23	0.90	(0.67, 1.22)	0.50	2.46	(1.09, 5.56)	0.03
Middle x Ado depression	2.02	(0.66, 6.20)	0.22	0.83	(0.34, 2.02)	0.68	2.00	(0.43, 9.22)	0.37
Sex	1.39	(0.88, 2.21)	0.16	0.62	(0.45, 0.85)	0.00*	0.64	(0.32, 1.30)	0.22
SES	0.62	(0.50, 0.77)	0.00*	1.28	(1.09, 1.50)	0.00*	0.41	(0.29, 0.60)	0.00*
Mother perceived impact	0.89	(0.80, 0.99)	0.04	-	-	-	-	-	-
Father perceived reactive hostility	0.87	(0.75, 1.00)	0.04	-	-	-	-	-	-
Mother ado. antisocial	-	-	-	1.26	(1.07, 1.48)	0.01*	-	-	-
Father perceived overprotection	-	-	-	0.92	(0.86, 0.98)	0.01*	-	-	-
Smoke pregnancy	-	-	-	-	-	-	2.66	(1.37, 5.18)	0.00*
Mother perceived self-efficacy	-	-	-	-	-	-	0.77	(0.62, 0.96)	0.05

*Factors remaining significant (<0.05) after applying Bonferroni correction

β were estimated with linear regressions, while OR were estimated with logistic regressions.
Abbreviation: OR = Odds ratio; CI = Confidence interval.

© 2024 Psychogiou L et al. *JAMA Network Open*.

eTable 4. Estimated Coefficients (β or OR) of Unadjusted and Adjusted Depression Symptoms at Every Time Point Associated With an Increased Risk of Reporting Impaired Adult Outcomes[a]

	**Outcomes**								
	**Depression**			**Social support**			**Perceived stress**		
Exposures (scores)	**beta**	**SD**	**p-value**	**beta**	**SD**	**p-value**	**beta**	**SD**	**p-value**
Early depression	0.01	0.12	0.92	0.10	0.30	0.73	-0.26	0.50	0.61
Middle depression	0.15	0.14	0.26	-1.60	0.33	<0.01	0.91	0.57	0.11
Early depression +	0.01	0.12	0.92	0.09	0.29	0.76	-0.25	0.50	0.62
Middle depression*	0.16	0.14	0.22	-1.60	0.33	<0.01	0.91	0.57	0.11
Adolescent depression	1.09	0.11	<0.01	-1.68	0.26	<0.01	4.50	0.44	<0.01
Early depression +	-0.01	0.12	0.90	0.14	0.29	0.64	-0.37	0.48	0.44
Middle depression +	0.09	0.14	0.50	-1.50	0.33	<0.01	0.61	0.55	0.27
Adolescent depression*	1.09	0.11	<0.01	-1.63	0.26	<0.01	4.49	0.44	<0.01

© 2024 Psychogiou L et al. *JAMA Network Open*.

	Outcomes								
	**Cybervictimization**			**NEET**			**Binge drinking**		
Exposures (scores)	**OR**	**95% CI**	**p-value**	**OR**	**95% CI**	**p-value**	**OR**	**95% CI**	**p-value**
Early depression	0.80	[0.54; 1.19]	0.27	1.00	[0.56; 1.79]	0.99	0.98	[0.74; 1.30]	0.74
Middle depression	1.22	[0.82; 1.83]	0.33	2.96	[1.76; 4.95]	<0.01	0.72	[0.52; 1.00]	0.06
Early depression +	0.80	[0.54; 1.19]	0.27	1.01	[0.56; 1.82]	0.97	0.97	[0.73; 1.29]	0.85
Middle depression*	1.22	[0.81; 1.82]	0.33	2.95	[1.76; 4.95]	<0.01	0.71	[0.52; 1.00]	0.06
Adolescent depression	1.79	[1.28; 2.52]	<0.01	1.66	[1.00; 2.77]	0.05	0.84	[0.66; 1.08]	0.18
Early depression +	0.80	[0.53;1.17]	0.24	1.00	[0.56; 1.81]	0.99	0.98	[0.74; 1.30]	0.88
Middle depression +	1.17	[0.78; 1.76]	0.44	2.88	[1.71; 1.81]	<0.01	0.72	[0.52; 1.00]	0.06
Adolescent depression*	1.79	[1.27; 2.52]	<0.01	1.60	[0.95; 2.67]	0.07	0.85	[0.66; 1.08]	0.19

[a]Data were compiled from the final master file of the Québec Longitudinal Study of Child Development (1998–2019), ©Gouvernement du Québec, Institut de la Statistique du Québec (Quebec Institute of Statistics).

* Adjusted models

© 2024 Psychogiou L et al. *JAMA Network Open*.
