# Supplementary material for: Childhood and Adolescent Depression Symptoms and Young Adult Mental Health and Psychosocial Outcomes
Source: JAMA Netw Open. 2024 Aug 8;7(8):e2425987. doi: 10.1001/jamanetworkopen.2024.25987 (PMC11310820; doi:10.1001/jamanetworkopen.2024.25987)
Supplement: Supplement 2. — Data Sharing Statement [file jamanetwopen-e2425987-s002.pdf]

Data Sharing Statement

Psychogiou. Childhood and Adolescent Depression Symptoms and Young Adult Mental Health and Psychosocial Outcomes. *JAMA Netw Open*. Published August 08, 2024.
doi:10.1001/jamanetworkopen.2024.25987

Data
Data available: No

Additional Information
Explanation for why data not available: The data used for this study are not owned by the authors. These data can be accessed by reaching out to the Institut de la Statistique du Québec (Quebec Institute of Statistics).
